# Identification of potential key genes for colorectal cancer based on bioinformatics analysis

**DOI:** 10.1097/MD.0000000000036615

**Published:** 2023-12-22

**Authors:** Chongyang Li, Shengqin Cao, Mingxiao Guo, Aihong Guo, Xuedi Sun

**Affiliations:** a Second Clinical Medical College, Binzhou Medical University, Yantai, China; b Jinan Fourth People’s Hospital, Jinan, China; c Department of General Surgery Center, Linyi People’s Hospital, Linyi, China; d Jinzhou Medical University, Jinzhou, China.

**Keywords:** bioinformatics analysis, biomarkers, CDK1, CEP55, colorectal cancer, MKI67, TOP2A

## Abstract

This study aimed to explore key genes as potential biomarkers for colorectal cancer (CRC) diagnosis and prognosis in order to improve their clinical utility. To identify and screen candidate genes involved in CRC carcinogenesis and disease progression, we downloaded the microarray datasets GSE143939, GSE196006, and GSE200427 from the GEO database and applied the GEO2R tool to obtain differentially expressed genes (DEGs) between colorectal cancer tissue samples and normal tissue samples. Differentially expressed genes were analyzed using the DAVID online database for gene ontology and Kyoto encyclopedia of genes and genomes pathway enrichment analyses. Protein-protein interaction network was constructed and related module analysis was performed using STRING and Cytoscape. In total, 241 DEGs were identified, including 127 downregulated and 114 upregulated genes. DEGs enriched functions and pathways included cellular response to chemical stimulus, extracellular region, carbonate dehydratase activity, cell division, spindle, and cell division. The abundant functions and pathways of DEGs included cellular response to chemical stimulus, extracellular region, carbonate dehydratase activity, cell division, spindle, cell adhesion molecule binding, Aldosterone-regulated sodium reabsorption, and Cell cycle-related processes. Fifteen key genes were identified, and bioprocess analyses showed that these genes were mainly enriched in cell cycle, cell division, mitotic spindle, and tubulin binding processes. It was found that CDK1, CEP55, MKI67, and TOP2A may be involved in CRC cancer invasion and recurrence. The pivotal genes identified in this study contribute to our understanding of the molecular and pathogenic mechanisms of CRC carcinogenesis and progression, and provide possible biomarkers for the diagnosis and treatment of CRC.

## 1. Introduction

Colorectal cancer (CRC) is one of the most common malignant tumors and has the second highest mortality rate and third highest incidence rate among cancers globally, leading to an estimated 1.9 million cases of morbidity and 900,000 deaths worldwide in 2020.^[1,2]^ According to a recent report published by GLOBOCAN 2020, the incidence of CRC is increasing in China^[3]^ Five-year survival prognosis greatly depends on the stage of the disease. While the survival rate of patients with stage I CRC is > 90%, that of patients with stage IV CRC is < 10%. The mainstay of treatment for CRC remains surgery, and postoperative chemotherapy for CRC, as one of the mainstays of postoperative treatment, has been used since the 1950s. Chemotherapy regimens based on the application of 5-fluorouracil remain the main treatment for CRC patients.^[4,5]^ With the development of science and technology, new chemotherapeutic agents such as capecitabine (Xeloda), oxaliplatin (Lexapro), and irinotecan (Kepto) have been added to the clinical practice.^[6]^ In terms of therapeutic strategies, clinical regimens such as cetuximab, bevacizumab, and monoclonal antibodies in combination with chemotherapy have also been added, and even though these regimens have improved the efficacy of the drugs against CRC, the 5-year survival rate of metastatic CRC is only 12%, and nearly half of the patients with metastatic CRC suffer from resistance to 5-fluorouracil-based therapy.^[7]^ In addition, gene therapy for translational purposes in the treatment of various cancers has made significant advances in the past decade. patients with CRC exhibit a variety of genetic changes and epigenetic modifications. The most common genetic alterations clinically associated with CRC are p53 and KRAS mutations. Gene therapy targeting defective genes such as TP53 (tumor suppressor gene encoding p53) and KRAS in CRC may serve as an alternative therapeutic pathway to standard treatment.^[8]^ In conclusion, personalized cancer drugs are becoming increasingly important in colorectal cancer treatment. Particularly for targeted therapies, there are large differences between individual treatment responses.^[9]^ Given the high morbidity and mortality of colorectal cancer, it is important and urgently needed to identify new biomarkers to unravel its pathogenesis, predict clinical outcomes, and enable personalized treatment for patients.

With the rapid development of cancer awareness and science and technology, microarray technology, bioinformatics, and clinical database analysis have been well-known and widely used for screening and identifying possible biomarkers in related cancers and applying them to clinical practice, which has led to a large number of differentially expressed genes (DEGs) being proved to have a close relationship with tumorigenesis and progression. Therefore, the screening and characterization of the associations and roles of the differentially expressed genes in CRC can provide a new biomarker for clinical treatment, which will offer a potential possibility to increase the cure rate of CRC and improve the quality of life of the patients.

## 2. Methods

The GEO database is a high-throughput public functional genomics gene expression database.^[10]^ We screened 3 gene expression datasets from the GEO database,^[11,12]^ including GSE143939, GSE196006, and GSE200427.The GSE143939 dataset contains 6 colorectal cancer tissue samples and 6 non-cancer tissue samples, and GSE196006 contains 21 colorectal cancer tissue samples and 21 non-cancer tissue samples. The GSE200427 dataset contains 2 colorectal cancer tissue samples and 2 non-cancer tissue samples.

The grouped analysis tool was analyzed in the GEO database using GEO2R, divided into cancerous tissue group and normal tissue group for analysis, and saved the analysis data in an Excel sheet. Differential genes required for this study were screened according to the adjusted *P* value of < .001 and logFC absolute value of more than 1.

A website (bioinformatics.psb.ugent.be/webtools/Venn/) was created through a VENN chart, and differential genes retrieved from an Excel spreadsheet that met the criteria were imported into the website. A VENN plot was created to identify differential genes co-expressed in the 3 gene expression datasets.

The Kyoto encyclopedia of genes and genomes (KEGG) and gene ontology (GO) enrichment analysis of co-expressed differential genes was performed through the DAVID Online Database (DAVID Functional Annotation Bioinformatics) (version 6.8). Microarray Analysis) (version 6.8). The DAVID online database provides users with gene and protein annotation information to extract biological data.^[13]^ KEGG is a genome deciphering database that includes complete and partially sequenced genome sequences, as well as information on metabolism, signaling, cell cycle, and membrane trafficking.^[14]^ Available information.^[14]^ GO is a major bioinformatics tool.^[15]^ GO categorizes gene functions into 3 parts: Cellular components (CC), molecular function (MF) and biological processes (BP). The GO database allows us to obtain annotation information for selected target genes in CC, MF and BP. Bioinformatics microarray analysis was performed using the DAVID online database to analyze the functions of the differential genes. *P* < .05 was statistically significant.

The protein-protein interaction network (PPI) network retrieves differential gene interactions through STRING (version: 11.5) (STRING: functional protein association networks). STRING online database allows for the analysis of protein interactions through module analysis and network construction.^[16]^ This helps us to delve deeper and investigate the mechanisms of events and the role of conditions. Cytoscape (version 3.9.0) is a bioinformatics software platform,^[17]^ and Cytoscape’s plugin molecular complex detection (MCODE) can be used for topology-based clustering of a given network to find densely connected regions.^[18]^ PPI networks are drawn using Cytoscape and the most critical modules in the PPI network are identified using MCODE. The selection criteria were as follows: MCODE score > 5, degree cutoff = 2, node score cutoff = 0.2, maximum depth = 100, and k-score = 2. The genes in the module were then subjected to KEGG and GO analyses using DAVID.

We analyzed gene networks and co-expressed genes using Network Analyst (version 3.0), a plugin that selects key genes for Cytoscape, with core genes having a rank of ≥ 10. The biological processes and associated functions of essential genes were visualized and analyzed through the Bio Network Gene Oncology plugin for Cytoscape.^[19]^ Gene classification was constructed by UCSC (UCSC Xena). Overall survival and disease-free survival of key genes were analyzed in cBioPortal using Kaplan–Meier curves.

Based on the gene expression profiling interactive analysis online database, we analyzed the expression differences of key genes between colorectal cancer samples and non-cancer tissue samples. The screening criteria were | Log2FC | cutoff: 1, *P* value cutoff: .01, jitter size: 0.4, matching TCGA criteria and GTEx data.

## 3. Results

**Figure 1.**
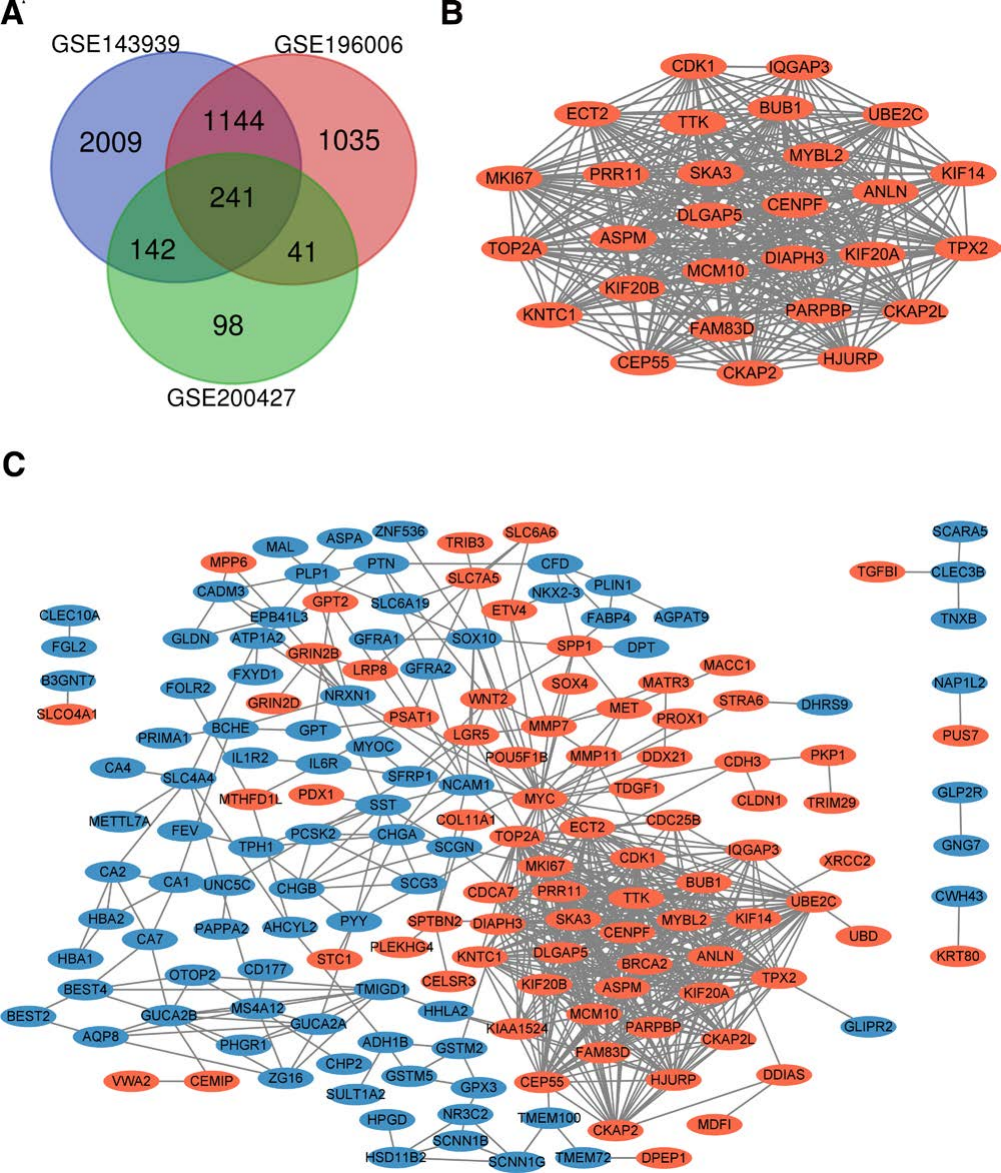
Venn diagram, PPI network, and the most significant module of DEGs. (A) DEGs were selected with a fold change > 2 and *P* value < .001 among the mRNA expression profiling sets GSE143939, GSE196006, and GSE200427. The three datasets showed an overlap of 241 genes. (B) The most significant module. (C) The PPI network of DEGs was constructed using Cytoscape. Upregulated genes are marked in light red; downregulated genes are kept in light blue. DEGs = differentially expressed genes, PPI = protein-protein interaction.

We screened 3 datasets related to colorectal cancer from the GEO database, and after unified processing of the relevant data in the dataset, common differentially expressed genes were identified and screened in the 3 datasets, of which GSE143939 contained 3536 genes, GSE196006 contained 2461 genes, and GSE200427 contained 522 genes. As shown in the VENN plot (Fig. 1), the overlap between the 3 datasets contained 241 genes, consisting of 127 downregulated genes and 114 upregulated genes between colorectal cancer tissues and non-cancer tissues.

KEGG and GO enrichment analysis of differential genes. To analyze the biological taxonomy of DEGs, we performed functional and pathway enrichment analysis using DAVID. GO analysis showed that the downregulated BPs of DEGs were mainly significantly enriched in cellular response to chemical stimulus, 1-carbon metabolic process, sodium ion transmembrane transport, cellular response to endogenous stimulus, response to steroid hormone, and response to endogenous stimulus were significantly enriched (Table 1). Changes in CC were significantly enriched mainly in the extracellular region, extracellular region part, external side of the plasma membrane, cell surface, and extracellular space, and MF was significantly enriched mainly in carbonate dehydratase activity. Upregulated DEGs BP were mainly enriched in cell division, cell cycle process, cell cycle, mitotic cell cycle, regulation of cell cycle process, regulation of cell cycle. Changes in cell composition (CC) were significantly enriched mainly in the spindle, spindle pole, mitotic spindle, and MF was significantly enriched mainly in cell adhesion molecule binding. KEGG pathway analysis showed that downregulated DEGs were mainly enriched in Aldosterone-regulated sodium reabsorption, Nitrogen metabolism, and Proximal tubule bicarbonate reclamation, while upregulated DEGs were mainly enriched in Cell cycle.

**Table 1:** GO and KEGG pathway enrichment analysis of DEGs in CRC samples.

Term	Description	Count in gene set	*P* value
Downregulated			
GO:0070887	Cellular response to chemical stimulus	38	1.14E-05
GO:0006730	One-carbon metabolic process	5	8.86E-05
GO:0035725	Sodium ion transmembrane transport	8	9.66E-05
GO:0071495	Cellular response to endogenous stimulus	21	1.13E-04
GO:0048545	Response to steroid hormone	10	1.97E-04
GO:0009719	Response to endogenous stimulus	23	3.51E-04
GO:0006814	Sodium ion transport	8	5.11E-04
GO:0019755	One-carbon compound transport	4	6.04E-04
GO:0098754	Detoxification	6	7.20E-04
GO:0030004	Cellular monovalent inorganic cation homeostasis	6	7.75E-04
GO:0005576	Extracellular region	57	5.68E-08
GO:0044421	Extracellular region part	48	1.09E-06
GO:0009897	External side of plasma membrane	12	2.23E-04
GO:0009986	Cell surface	18	2.96E-04
GO:0005615	Extracellular space	27	3.30E-04
GO:0004089	Carbonate dehydratase activity	4	1.60E-04
hsa04960	Aldosterone-regulated sodium reabsorption	5	9.65E-05
hsa00910	Nitrogen metabolism	4	1.75E-04
hsa04964	Proximal tubule bicarbonate reclamation	4	4.42E-04
Upregulated			
GO:0051301	Cell division	23	5.45E-13
GO:0022402	Cell cycle process	29	1.13E-11
GO:0007049	Cell cycle	33	3.77E-11
GO:0000278	Mitotic cell cycle	25	3.95E-11
GO:0010564	Regulation of cell cycle process	22	4.60E-11
GO:0051726	Regulation of cell cycle	26	4.66E-10
GO:0000280	Nuclear division	18	1.12E-09
GO:0008283	Cell proliferation	33	1.36E-09
GO:0005819	Spindle	17	1.08E-09
GO:0000922	Spindle pole	10	3.77E-07
GO:0072686	Mitotic spindle	10	6.55E-07
GO:0015630	Microtubule cytoskeleton	22	1.15E-05
GO:0005694	Chromosome	25	3.95E-05
GO:0044427	Chromosomal part	23	7.40E-05
GO:0005813	Centrosome	14	9.53E-05
GO:0005815	Microtubule organizing centre	15	1.82E-04
GO:0050839	Cell adhesion molecule binding	11	4.01E-04
hsa04110	Cell cycle	5	0.006193153

CC = colorectal Cancer, CRC = colorectal cancer, DEGs = differentially expressed genes, GO = Gene Ontology, KEGG = Kyoto Encyclopedia of Genes and Genomes.

PPI network structure and module analysis. The PPI network diagram of DEGs was constructed (Fig. 1B) and the most important modules were obtained using Cytoscape (Fig. 1C). The genes involved in this module were functionally analyzed using DAVID technology. The results showed that the genes in this module were mainly enriched in cell cycle, cell division, spindle, microtubule organizing center, microtubule, and Motor proteins (Table 2).

**Table 2:** GO and KEGG pathway enrichment analysis of DEGs in the most powerful module.

Pathway ID	Pathway description	Count in gene set	FDR
GO:0007049	Cell cycle	23	7.01E-17
GO:0051301	Cell division	18	7.01E-17
GO:0022402	Cell cycle process	21	1.19E-16
GO:0000278	Mitotic cell cycle	19	9.29E-16
GO:1903047	Mitotic cell cycle process	17	5.96E-14
GO:0000280	Nuclear division	15	1.58E-13
GO:0048285	Organelle fission	15	3.84E-13
GO:0010564	Regulation of cell cycle process	15	7.41E-12
GO:0051726	Regulation of cell cycle	17	1.29E-11
GO:0007059	Chromosome segregation	12	9.25E-11
GO:0005819	Spindle	15	2.06E-14
GO:0072686	Mitotic spindle	10	1.98E-10
GO:0015630	Microtubule cytoskeleton	17	2.26E-10
GO:0000922	Spindle pole	9	2.85E-09
GO:0030496	Midbody	9	9.26E-09
GO:0005815	Microtubule organizing centre	11	3.58E-06
GO:0005874	Microtubule	9	3.88E-06
GO:0005813	Centrosome	10	6.06E-06
GO:0099513	Polymeric cytoskeletal fiber	10	1.55E-05
GO:0008017	Microtubule binding	7	1.66E-04
GO:0008092	Cytoskeletal protein binding	10	2.42E-04
GO:0015631	Tubulin binding	7	4.10E-04
hsa04110	Cell cycle	3	0.123791
hsa04814	Motor proteins	3	0.123791

DEGs = differentially expressed genes, FDR = false discovery rate, GO = Gene ontology, KEGG = Kyoto encyclopedia of genes and genomes.

**Figure 2.**
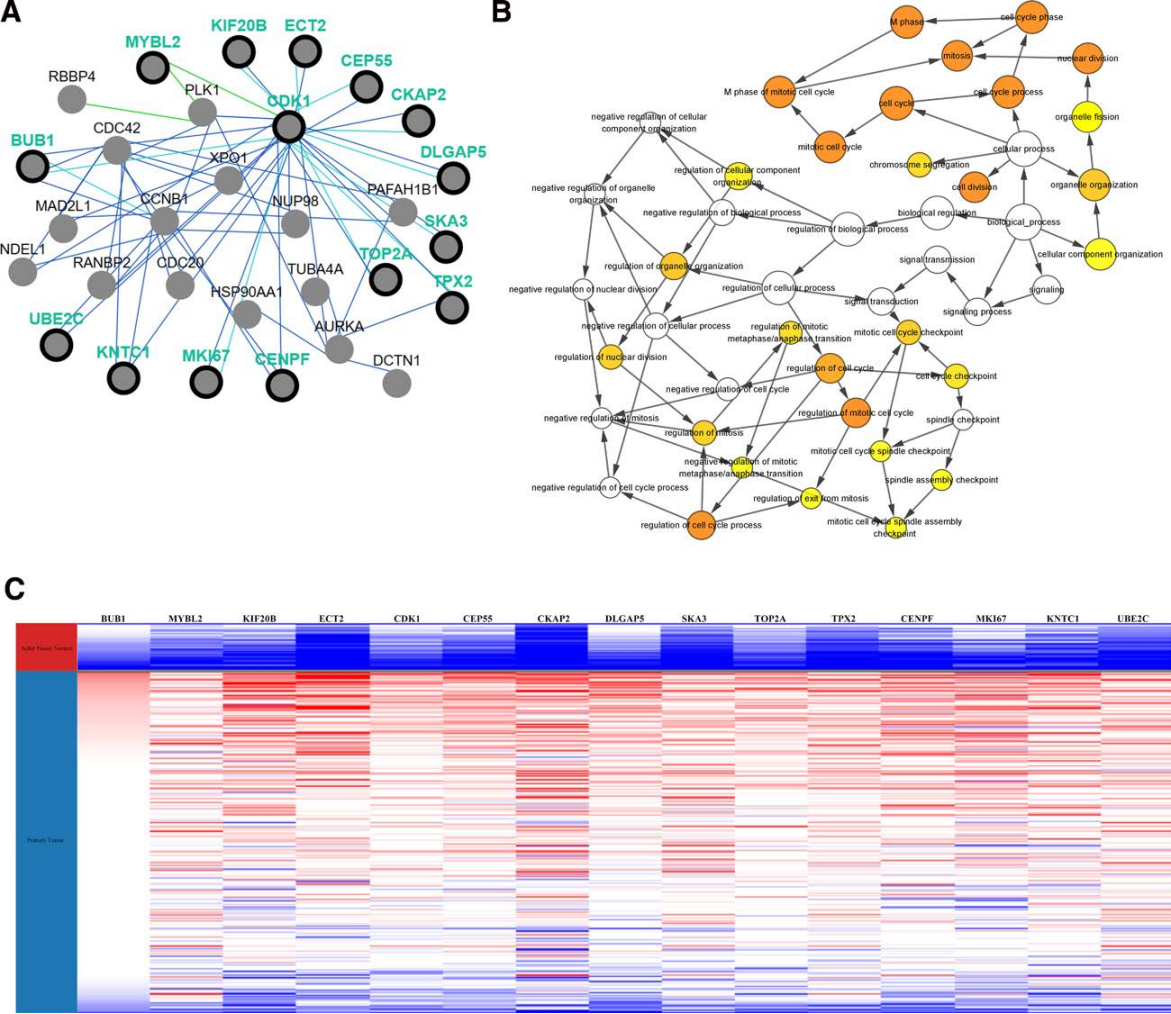
Interaction network and biological process analysis of the hub genes. (A) Hub genes and their co-expression genes were analyzed using PCViz (pathway commons networks visualizer). Nodes with bold black outlines represent hub genes. Nodes with gray represent the co-expression genes. (B) The biological process analysis of hub genes was constructed using BiNGO. The color depth of nodes refers to the corrected *P* value of ontologies. The size of nodes refers to the number of genes involved in the ontologies. *P* < .01 was considered statistically significant. (C) The hierarchical clustering of hub genes was constructed using UCSC. The samples under the red bar are noncancerous, and the samples under the blue bar are HCC samples. The upregulation of genes is marked in red; the downregulation of genes is marked in blue; null is marked in gray.

**Figure 3.**
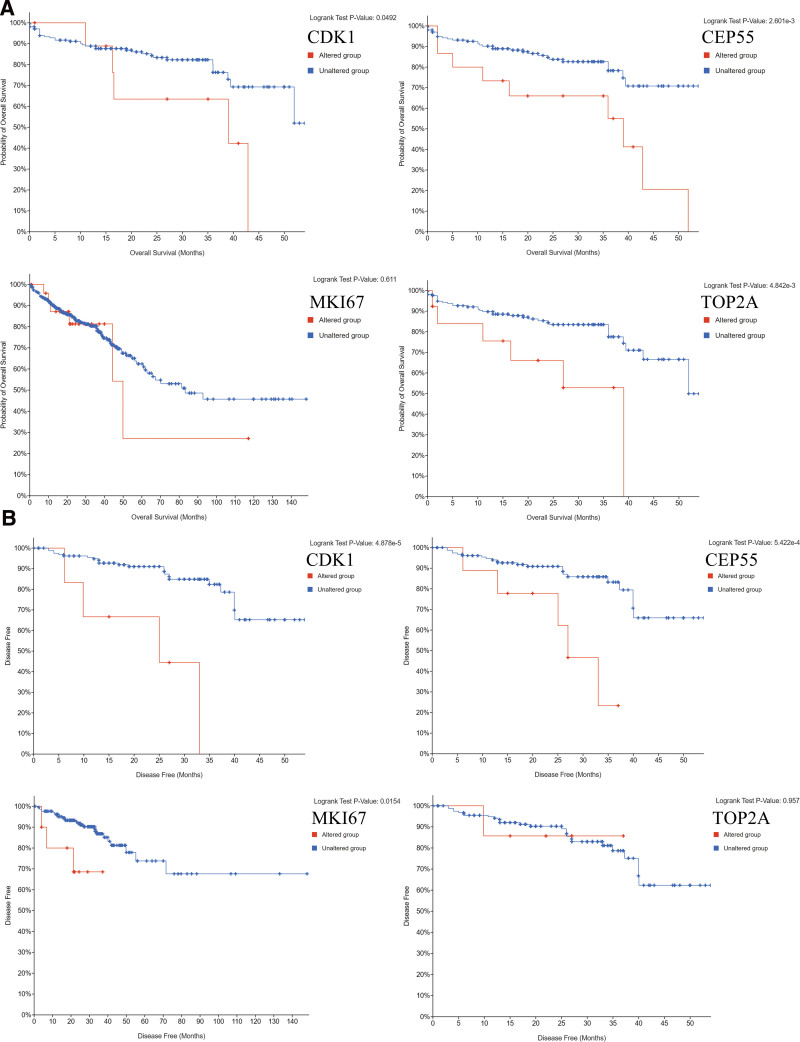
(A) Overall survival and (B) disease-free survival analyses of hub genes were performed using the cBioPortal online platform. *P* < .05 was considered statistically significant. Red cases represent the cases with alterations in query genes. Blue cases represent the cases without alterations in query genes.

Selection and analysis of key genes. A total of 15 genes were identified as central genes with degree ≥ 10. The names, abbreviations and functions of these central genes are shown in Table 3. The network of central genes and their co-expressed genes were analyzed using the cBioPortal online platform (Fig. 2A). The biological processes of the key genes were analyzed as shown in Figure 2B. Hierarchical clustering results showed that the key genes could basically distinguish hepatocellular carcinoma samples from noncancerous samples (Fig. 2C). Subsequently, the overall survival of the key genes was analyzed using Kaplan–Meier curves. Overall survival of CRC patients with alterations in CDK1, TOP2A, and CEP55 was poor (Fig. 3A). Nevertheless, CRC patients with CDK1, MKI67, and CEP55 alterations had poorer disease-free survival (Fig. 3B). In addition, MKI67 alterations were not associated with overall survival, but were significantly associated with poorer disease-free survival (*P* = .0611 for overall survival and *P* = .0154 for disease-free survival). In contrast, TOP2A alterations were significantly associated with poorer overall survival, but not with disease-free survival (overall survival *P* = 4.842E-3, disease-free survival *P* = .957).

**Table 3:** Functional roles of 20 hub genes with degree ≥ 10.

Gene Symbol	Full name	Function
BUB1	BUB1 mitotic checkpoint serine/threonine kinase	This gene encodes a serine/threonine-protein kinase that play a central role in mitosis. The encoded protein functions in part by phosphorylating members of the mitotic checkpoint complex and activating the spindle checkpoint
MYBL2	MYB proto-oncogene like 2	The protein encoded by this gene, a member of the MYB family of transcription factor genes, is a nuclear protein involved in cell cycle progression. The encoded protein is phosphorylated by cyclin A/cyclin-dependent kinase 2 during the S-phase of the cell cycle and possesses both activator and repressor activities
KIF20B	Kinesin family member 20B	Enables several functions, including WW domain binding activity; plus-end-directed microtubule motor activity; and protein homodimerization activity
ECT2	Epithelial cell transforming 2	The protein encoded by this gene is a guanine nucleotide exchange factor and transforming protein that is related to Rho-specific exchange factors and yeast cell cycle regulators. The expression of this gene is elevated with the onset of DNA synthesis and remains elevated during G2 and M phases
CDK1	Cyclin-dependent kinase 1	The protein encoded by this gene is a member of the Ser/Thr protein kinase family. This protein is a catalytic subunit of the highly conserved protein kinase complex known as M-phase promoting factor (MPF), which is essential for G2/M phase transitions of eukaryotic cell cycle
CEP55	Centrosomal protein 55	Enables identical protein binding activity. Involved in cranial skeletal system development; establishment of protein localization; and midbody abscission. Acts upstream of or within mitotic cytokinesis
CKAP2	Cytoskeleton-associated protein 2	This gene encodes a cytoskeleton-associated protein that stabalizes microtubules and plays a role in the regulation of cell division.
DLGAP5	DLG associated protein 5	Predicted to enable microtubule binding activity. Predicted to be involved in several processes, including centrosome localization; kinetochore assembly; and mitotic spindle organization.
SKA3	Spindle and kinetochore associated complex subunit 3	This gene encodes a component of the spindle and kinetochore associated protein complex that regulates microtubule attachment to the kinetochores during mitosis.
TOP2A	DNA topoisomerase II alpha	This gene encodes a DNA topoisomerase, an enzyme that controls and alters the topologic states of DNA during transcription.
TPX2	TPX2 microtubule nucleation factor	Enables importin-alpha family protein binding activity and protein kinase binding activity. Involved in activation of protein kinase activity; microtubule cytoskeleton organization; and negative regulation of microtubule depolymerization.
CENPF	Centromere protein F	This gene encodes a protein that associates with the centromere-kinetochore complex. The protein is a component of the nuclear matrix during the G2 phase of interphase. In late G2 the protein associates with the kinetochore and maintains this association through early anaphase.
MKI67	Marker of proliferation Ki-67	Enables protein C-terminus binding activity. Involved in regulation of chromosome segregation and regulation of mitotic nuclear division.
KNTC1	Kinetochore associated 1	This gene encodes a protein that is one of many involved in mechanisms to ensure proper chromosome segregation during cell division.
UBE2C	Ubiquitin conjugating enzyme E2 C	The modification of proteins with ubiquitin is an important cellular mechanism for targeting abnormal or short-lived proteins for degradation.

**Figure 4.**
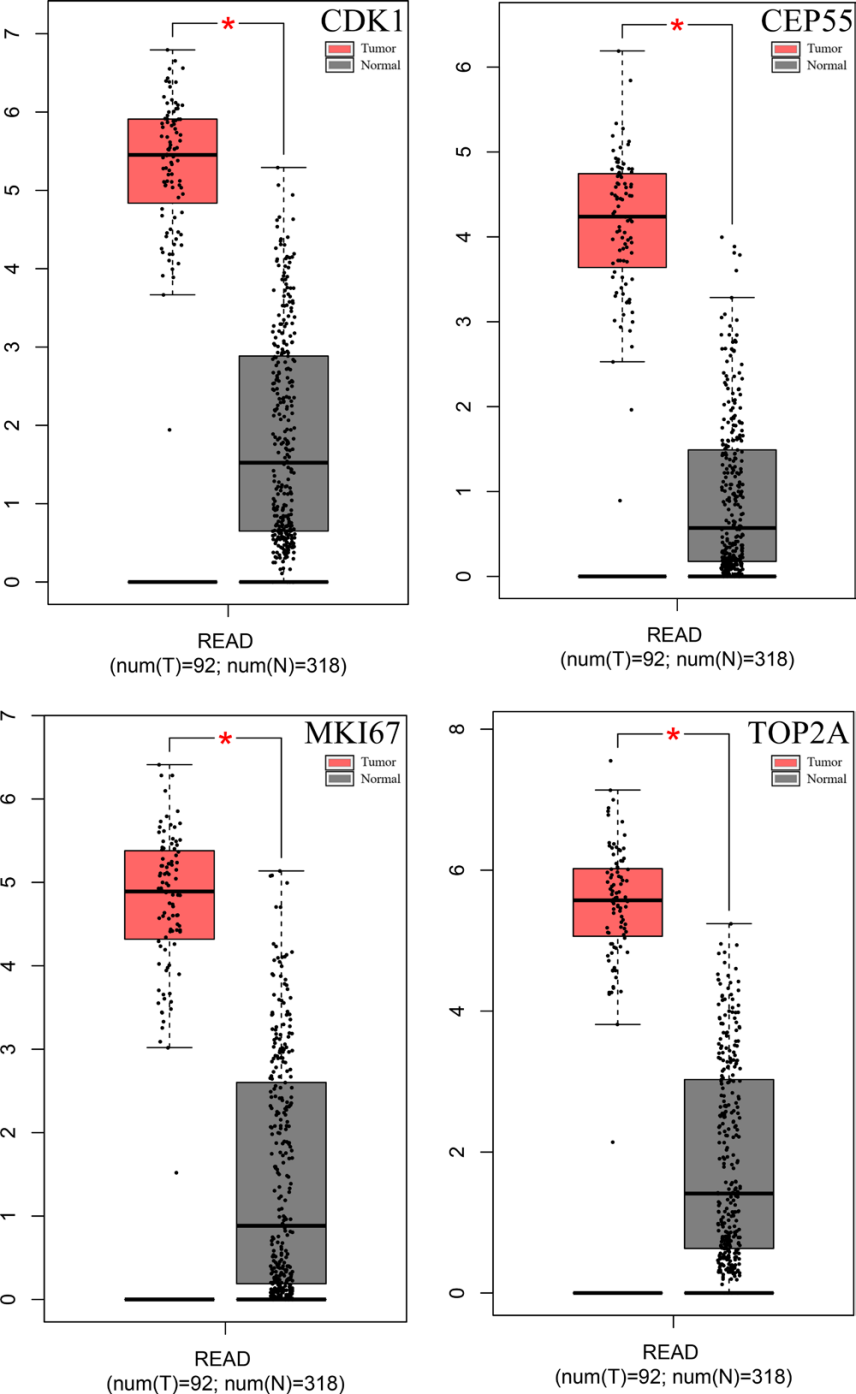
mRNA expression level of CDK1, CEP55, MKI67, and TOP2A based on TCGA data by GEPIA. The expression levels of CDK1, CEP55, MKI67, and TOP2A between READ and the normal samples were consistent with the results of GEO. Log scale: We use log2 (TPM + 1) for log-scale. |Log_2_FC| Cutoff: 1; *P* value Cutoff: 0.01. COAD = colon adenocarcinoma, GEO = gene expression omnibus, GEPIA = gene expression profiling interactive analysis, TCGA = the cancer genome atlas, TPM = transcripts per kilobase of exon model per million mapped reads.

**Figure 5.**
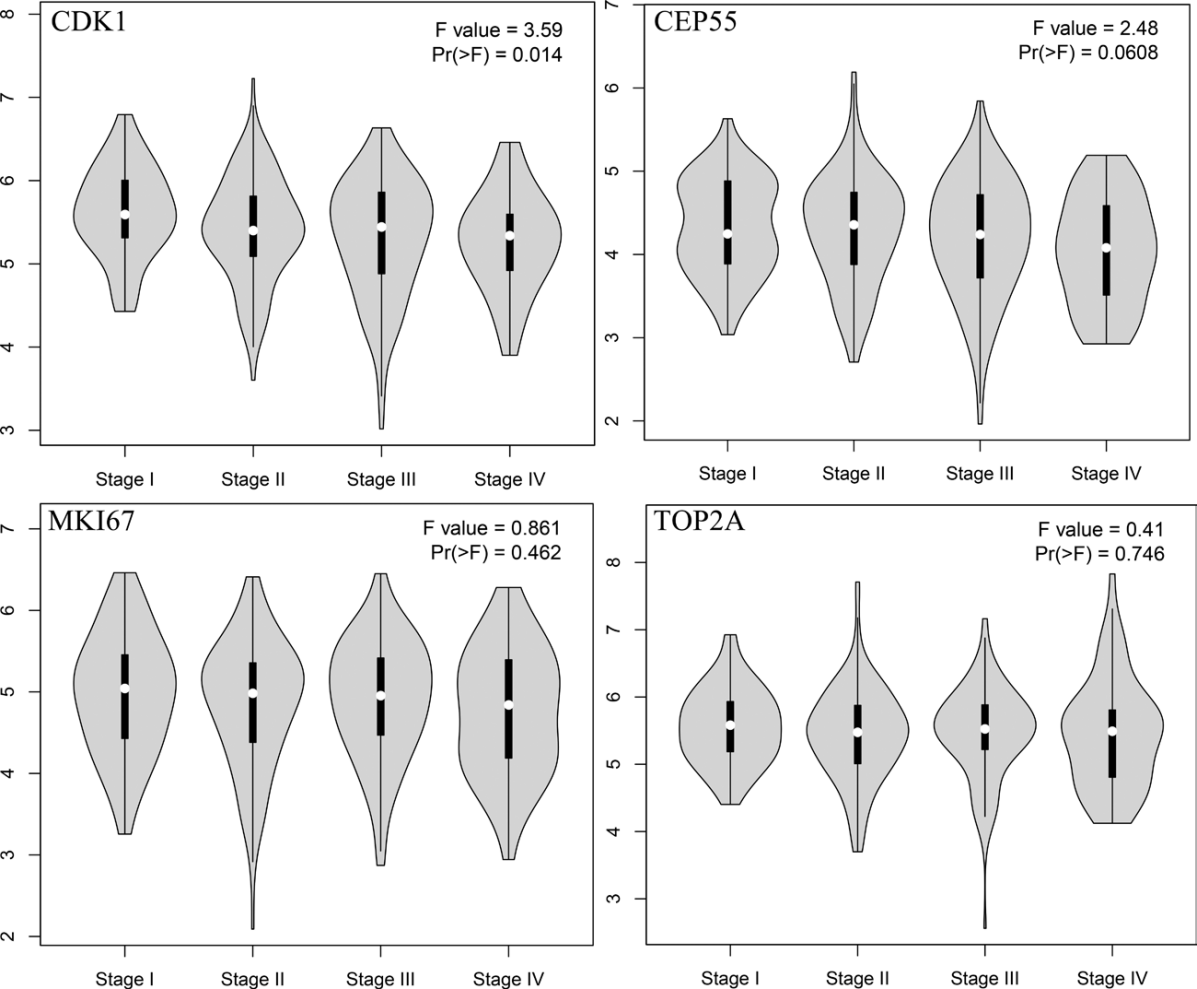
The mRNA expression level of CDK1, CEP55, MKI67, and TOP2A in tumor grade based on TCGA data by GEPIA. GEPIA = gene expression profiling interactive analysis.

Subsequently, we analyzed the expression of these 4 genes in tumor tissues and normal tissues (Fig. 4) and the changes in tumor grade (Fig. 5) by the gene expression profiling interactive analysis online database. The expression of CDK1, MKI67, TOP2A, and CEP55 was significantly higher in tumor tissue than in normal tissue. The changes in CDK1 in different grades of colon cancer tissues were also statistically significant (Pr ( > F) = 0.014). CDK1 expression in different graded colon cancer tissues was also statistically significant (Pr ( > F) = 0.014). The changes in CEP55, MKI67, and TOP2A were not statistically significant.

## 4. Discussion

CRC is one of the most common cancers with a high degree of malignancy and ranks equally high in malignancy-related mortality.^[3]^ Despite promising advances in the treatment of CRC, the metastasis and recurrence rates of CRC are high, and the survival prognosis of CRC patients remains poor.^[20]^ Therefore, further understanding of the etiology and molecular mechanisms of CRC can provide a large number of potential possibilities for the development of novel therapeutic agents and treatments.

In this study, we extracted gene expression datasets from the GEO database to identify potential key genes associated with CRC. A total of 127 downregulated genes and 114 upregulated genes were identified by using GEO2R to detect DEGs in colorectal cancer tissue samples versus normal tissue samples. These DEGs were shown to be mainly involved in GO enrichment analysis of cellular response to chemical stimulus, extracellular region, carbonate dehydratase activity, cell division, spindle, cell adhesion molecule binding, Aldosterone-regulated sodium reabsorption for KEGG pathway analysis, Cell cycle, etc, and is in line with our knowledge,^[21]^ such as Jakub Styk et al^[22]^ found that the presence of free nucleic acids in the cycle (cfNAs) present in the circulation are bound to protein complexes or encapsulated in extracellular membrane vesicles (e.g., apoptotic vesicles, microvesicles, or exosomes), which mediate intercellular communication and thus play an important role in the pathogenesis of colorectal cancer. It has been suggested that low carbonic anhydrase I and carbonic anhydrase II hydratase activities and a corresponding decrease in HCO_3_^−^ secretion in CRC-infiltrated T lymphocytes may indicate a dysregulation of the local immune response and a consequent loss of effective anticancer mechanisms.^[23]^ In conclusion the factors revealed by GO enrichment analysis corroborate with numerous studies, suggesting that these factors are critical for the development and progression of CRC.

We assessed the association of DEGs by PPI network and module analyses, and a total of 15 key genes were screened and identified, including BUB1, MYBL2, kinesin family member 20B (KIF20B), ECT2, CDK1, CEP55, CKAP2, DLGAP5, SKA3, TOP2A, TPX2, CENPF, MKI67, KNTC1, and UBE2C. In a Kaplan–Meier survival analysis, overall survival was found to be worse for highly expressed CDK1, CEP55 and TOP2A, and disease-free survival was found to be worse for highly expressed CDK1, CEP55 and MKI67. Suggesting that high expression of CDK1, CEP55, MKI67, and TOP2A are poor prognostic factors for CRC patients.

CDK1 is a member of the Ser/Thr family of protein kinases, which are the catalytic subunits of highly conserved protein kinase complexes known as M-phase promoters, and are essential for the G2/M-phase transition of the eukaryotic cell cycle. The fervor about the association between CDK1 and CRC has continued in recent years, and it has been shown that dihydroartemisinin can inhibit CRC genesis and cell cycle by targeting CDK1/CCNB1/PLK1 signaling.^[24]^ Dipeptidyl peptidase 3 is a zinc-dependent hydrolase, and dipeptidyl peptidase 3 can reduce the tumourigenicity of CRC cells in vivo by targeting CDK1, which in turn can have an effect on CRC cell function.^[25]^ During mitosis, CDK1 plays a largely regulatory role and the process is also regulated by CDK1 to achieve autophagy when it occurs in cells.^[26]^ CEP55 was originally identified as a key component of abscission, which is the final stage of cell division, and is used to regulate the physical separation of the 2 daughter cells.^[27]^ Huang RH et al^[28]^ observed immunohistochemically that 55 cancer specimens were from colorectal cancer patients and found that CEP55 overexpression predicted good prognosis in patients with lymph node metastasis from colorectal cancer. And Cep55/c10orf3-derived peptide vaccine was found to be feasible for the treatment of colorectal cancer in related experiments.^[29]^ MKI67 is mainly involved in the regulation of chromosome segregation and mitotic nuclei, and it has been found that knockdown of Pleckstrin homology-like domain family A member 2 suppressed the tumourigenesis and expression of KI67 protein in CRC patients.^[30]^ On the other hand, TOP2A, as an enzyme that controls and alters the topological state of DNA during transcription, high expression of TOP2A promotes the malignant progression of lung, liver, breast, and colorectal cancers, suggesting a poor prognosis.^[31–34]^ In a related study of CRC, the knockdown of TOP2A was found to significantly promote the growth and proliferation rate of CRC cells.^[35]^ On the other hand, germline mutations in BUB1 increase the risk of CRC at a young age.^[36]^ MYBL2 is an independent prognostic marker with tumor-promoting function in CRC and MYBL2 is overexpressed in CRC and therefore may play an important role in tumourigenesis.^[37]^ KIF20B is oncogenic in bladder and hepatocellular carcinoma cells, but its role in CRC progression is not well understood, Lin WF et al^[38]^ demonstrated experimentally that KIF20B overexpression promotes the glioma-associated oncogene 1-mediated epithelial-mesenchymal transition process and CRC cell migration and invasion.ECT is essential for cell division, and Cook DR et al^[39]^ found that ECT2 overexpression and mislocalisation support its role as a driver of colon cancer, independent of its function in normal cell division. DLGAP5 can be involved in aspects of centrosome localization, kinetochore assembly, and mitotic spindle organization. It has been confirmed that DLGAP5 has a very important role in determining the aggressiveness of various CRC phenotypes.^[40]^ In addition, the development of CRC involves the sequential transformation of normal mucosal tissues into benign adenomas and ultimately into malignant tumors in the intestinal tract, and SKA3 at chromosome 13q has been identified as a novel gene involved in the promotion of malignant transformation, and it has been found that knockdown of SKA3 in CRC cells induces G2/M arrest and reduces migration, invasion and anchorage-independent growth.^[41]^ Similarly, TPX2-promoted 20q amplicon drive likewise allows colorectal adenomas to progress to intestinal tumors.^[42]^ On the other hand, Gastrointestinal stromal tumors are considered to be tumors with relatively poor prognosis and Chen WB et al^[43]^ found CENPF to be a marker of malignant behavior in CRGIST. expression of CENPF indicates poor clinical outcome, so CENPF expression may be a potential therapeutic strategy for the treatment of malignant CRGIST. Also in colorectal cancer tissues, KNTC1 is largely highly expressed and correlates with pathological grading of the disease and overall survival. Knockdown of KNTC1 inhibits colorectal cancer cell proliferation, cell cycle, migration and in vivo tumourigenesis.^[44]^ However, the role of UBE2C in CRC is not well defined and further studies are needed.

## 5. Conclusion

This study aimed to investigate differential genes that may be involved in colorectal carcinogenesis and progression. 241 differential genes and 15 essential genes (degree ≥ 10) have been identified. These genes may be identified as biomarkers of cancer and provide new insights into the diagnosis and treatment of CRC, but their specific biological functions and significance require further study.

## Acknowledgments

This study was supported by the National Natural Science Foundation of China (Grant no.81500688) and Shandong Provincial Natural Science Foundation (ZR2021MH362).

## Author contributions

**Conceptualization:** Chongyang Li, Xuedi Sun.

**Data curation:** Chongyang Li, Shengqin Cao, Xuedi Sun.

**Formal analysis:** Chongyang Li, Xuedi Sun.

**Funding acquisition:** Chongyang Li, Mingxiao Guo.

**Investigation:** Chongyang Li, Shengqin Cao.

**Methodology:** Chongyang Li.

**Project administration:** Chongyang Li.

**Resources:** Chongyang Li.

**Software:** Chongyang Li, Aihong Guo.

**Supervision:** Chongyang Li, Shengqin Cao.

**Validation:** Chongyang Li.

**Visualization:** Chongyang Li, Shengqin Cao.

**Writing – original draft:** Chongyang Li.

**Writing – review & editing:** Chongyang Li.
